# Relevance of tissue Doppler in the quantification of stress echocardiography for the detection of myocardial ischemia in clinical practice

**DOI:** 10.1186/1476-7120-3-2

**Published:** 2005-01-28

**Authors:** Rosa Sicari

**Affiliations:** 1 Department of Echocardiography, Institute of Clinical Physiology, National Council of Research, Pisa, Italy

## Abstract

In the present article we review the main published data on the application of Tissue Doppler Imaging (TDI) to stress echocardiography for the detection of myocardial ischemia. TDI has been applied to stress echocardiography in order to overcome the limitations of visual analysis for myocardial ischemia. The introduction of a new technology for clinical routine use should pass through the different phases of scientific assessment from feasibility studies to large multicenter studies, from efficacy to effectiveness studies. Nonetheless the pro-technology bias plays a major role in medicine and expensive and sophisticated techniques are accepted before their real usefulness and incremental value to the available ones is assessed. Apparently, TDI is not exempted by this approach : its applications are not substantiated by strong and sound results. Nonetheless, conventional stress echocardiography for myocardial ischemia detection is heavily criticized on the basis of its subjectivity. Stress echocardiography has a long lasting history and the evidence collected over 20 years positioned it as an established tool for the detection and prognostication of coronary artery disease. The quantitative assessment of myocardial ischemia remains a scientific challenge and a clinical goal but time has not come for these newer ultrasonographic techniques which should be restricted to research laboratories.

## 

Pharmacologic stress echocardiography is an established cost-effective technique for the detection of coronary artery disease [1]. The widespread use in the clinical practice has become possible only after evidence collected through large scale multicenter studies that demonstrated its feasibility, safety, diagnostic and prognostic accuracy [4-8]. According to the guidelines of ACC/AHA – pharmacological stress echocardiography with either dobutamine or dipyridamole is a class I indication (of documented effectiveness and usefulness) for the diagnosis of coronary artery disease and for the prognostic stratification of patients with known coronary artery disease [2,3]. Its major limitation is related to a high inter-observer variability and to operator-dependent expertise that might be overcome by an appropriate training and the use of strict reading criteria [9-11]. Nonetheless the hunt for an objective, operator-independent technique to be applied to the conventional black and white regional wall motion analysis remains a major goal in stress echocardiography. Tissue Doppler Imaging (TDI) provides a quantitative analysis of regional myocardial function through the analysis of myocardial velocities [12,13]. Since velocity imaging is confounded by influence from velocities in other segments, the TDI – based modalities strain and strain rate imaging have been introduced to measure regional shortening fraction and shortening rate, respectively [14] Is the application of Tissue Doppler Imaging to stress echocardiography the technique that will solve it all? According to major journals the answer is yes: the diagnostic accuracy of stress echocardiography improves with TDI when analyzed in comparison with visual assessment of wall motion analysis for the detection of inducible ischemia. Inducible ischemia quantified in a number without the approximations of visual assessment. However the enthusiasm showed by some investigators is not substantiated by scientific results. In fact, a careful analysis of the data published so far raises more doubts than certainties.

## What we talk about when we talk about TDI

The TDI modalities include myocardial velocity imaging, displacement imaging, strain rate imaging and strain imaging. TDI measures velocities by the Doppler shift of reflected ultrasound. Velocities are measured in the conventional imaging planes, from apical views as longitudinal velocities and from parasternal views as radial velocities. When we employ TDI, the velocities within a myocardial segment are the net result of motion caused by contractions in that segment, motion due to tethering to other segments, and overall motion of the heart. This tethering effect is the reason why longitudinal velocities increase progressively from the apex toward the base, when measured in an apical window. Therefore ischemia in the apical region reduces myocardial velocities not only in the apex, but also in the nonischemic basal segments [15]. In practical terms, the reduction of TDI velocities in basal segments is not synonymous of reduction of function in the same segments. The opposite effect, tethering of nonischemic segments might induce increase in velocity of adjacent ischemic segments. These limitations could be overcome by the employment of strain and strain rate. Strain rate reflects how fast regional myocardial shortening or lengthening occurs measured at two locations separated by a distance. This is the reason why some authors define strain rate as a spatial velocity gradient. Strain is calculated as the time integral of strain rate and is a dimensionless quantity. The limitations of TDI have been widely outlined [16-18] and this is beyond the scope of the present review but they may be synthesized into two main problems: 1 – the amplitude of the estimated velocity is dependent on the angle at which the region is imaged; 2 – the overall cardiac motion, rotation, and contraction of adjacent segments will influence regional velocity estimates. Therefore, a more critical approach to this technology would have avoided the inconsistencies of scientific results when it was applied in the clinical arena.

## TDI and Stress echocardiography for myocardial ischemia detection

Feasibility studies have been published demonstrating the applicability of TDI to stress echocardiography [19-27] but only few studies addressed the issue of its diagnostic accuracy in a clinical context [28-31].

Cain et al [28] applied myocardial Doppler velocity to dobutamine stress echocardiography in order to assess its diagnostic accuracy when compared to conventional visual assessment. They first identified the normality ranges of myocardial velocities in patients with normal coronary arteries or with a very low pretest probability of having coronary artery disease. Then they selected 114 patient with coronary artery disease assessed at coronary angiography and evaluated the diagnostic accuracy: see Table 1 (Additional file 1). Neither overall nor vascular territory accuracy was better for myocardial velocity when compared to visual wall motion scoring. The MYDISE Study [29] was the first multicenter study on the absolute value of TDI applied to dobutamine stress echocardiography. The study enrolled 289 patients separated in 3 groups: group 1 (n = 92) healthy volunteers or patients with normal coronary arteries, group 2 (n = 48) patients with known coronary artery disease and group 3 (n = 149) consecutive patients with known or suspected coronary artery disease. Exclusion criteria were: atrial fibrillation, previous myocardial infarction (Q waves on the electrocardiogram, or akinetic segments on the resting echocardiographic images), previous revascularization, unstable angina, complete bundle branch block, significant heart valve disease, contraindication to dobutamine or atropine). The diagnostic criteria were developed by comparing 92 normal subjects with 48 patients with coronary artery disease and applied in a prospective series of 149 patients referred to stress echo laboratory for the suspect of coronary artery disease. Velocity cut-off points were tested and discarded since they did nor perform well when compared to logistic regression models, using systolic velocities at peak stress in 7 myocardial segments and after adjusting for heart rate, age and gender [29].

**Figure 1:**
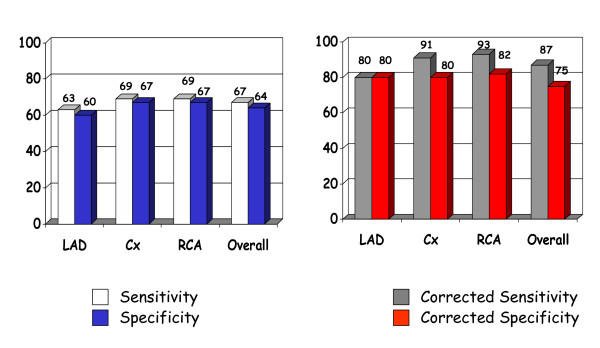
In the left panel sensitivities and specificities in the three main vascular districts and overall sensitivities and specificities without correcting results for age, gender and heart rate. In the right panel, the same data corrected for age, gender and heart rate (modified from ref.29).

**Figure 2:**
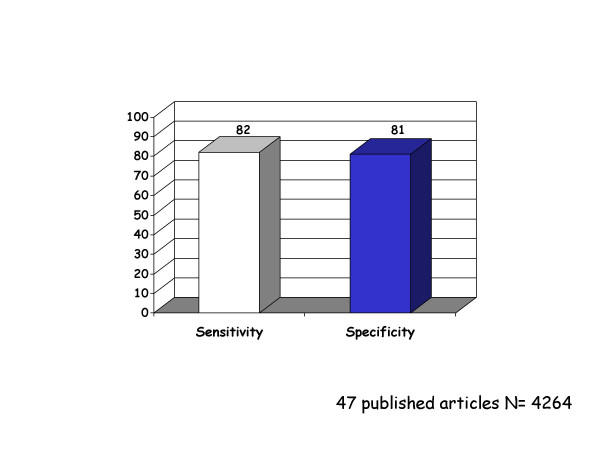
Sensitivity and specificity of dobutamine stress echocardiography

The main concerns refer to strict stress echocardiographic issues:1.the lack of a comparison between conventional visual assessment of regional wall motion and TDI analysis. In absolute terms the diagnostic accuracy is not striking: see Table 1 (Additional file 1) and Fig 1. Even if we accept the hypothesis of a non-inferiority analysis of TDI versus dobutamine stress echocardiography we have to take into consideration some major limitations outlined by the authors: the optimal diagnostic accuracy was obtained by using peak systolic velocity after adjusting for maximal heart rate, age and gender: "ignoring these factors reduces both sensitivity and specificity" [29]. Moreover, authors applied a very complex regression model for diagnostic accuracy assessment. A recent meta-analysis on dobutamine stress echocardiography showed an overall sensitivity of 80% and a specificity of 87% [32] (see fig 2). 2 – The extent and severity of myocardial ischemia as defined by the number of ischemic segments and the pharmacologic load is never provided. The protocol was interrupted only in the presence of secondary criteria, but never for development of myocardial ischemia since the quantitative analysis was performed off-line. It has been demonstrated that diagnostic and prognostic accuracy of stress echocardiography increases when the response is stratified in the time and space domain, i.e. number of ischemic segments, severity of ischemia induced, the time of onset of ischemia and the pharmacologic dose. 3 – the apical segments have been excluded by the analysis since the systolic velocity is not reliable making the analysis possible only in 11 segments. Nonetheless, the apex and the apical segments are very often the site of stress echocardiographic positivity unless very proximal atherosclerotic lesions are present 4 – the time for performing analysis is never reported. We are informed that the comparison between systolic velocities at rest and at peak stress is disregarded since it is time consuming and increases the potential for observer variability without increasing diagnostic accuracy. 5 -, apparently, TDI cannot be applied to patients with wall motion abnormalities at rest. The exclusion of this group of patients makes this quantitative approach quite unfeasible for routine clinical application.

Voigt et al. [30] used a more realistic approach to the application of TDI to stress echocardiography. They first demonstrated in 44 patients with known or suspected coronary artery disease that strain rate quantitatively differentiates ischemic and nonischemic regional myocardial response to dobutamine stress echocardiography [30] and compared it with conventional visual assessment. The ratio of postsystolic shortening to maximal strain was the best quantitative parameter to identify dobutamine stress induced-ischemia. This quantitative analysis improved sensitivity from 81 (visual assessment) to 86% and specificity from 82% to 89%. The statistical significance is not provided in the manuscript. Then, in the same population of 44 [31], they compared the visual assessment of wall motion abnormalities with different parameters derived from TDI application such as peak systolic velocity, systolic displacement and strain rate imaging. They employed simultaneous perfusion scintigraphy as a gold standard of myocardial ischemia. The stress echocardiographic methodology employed was not a standard one for segmentation of the left ventricle (18 segments instead of 16 or 17), pharmacologic protocol (up to 2 mg atropine instead of 1 mg) and criteria for ischemia (worsening of wall motion only in 1 segment). Also in this case, the overall accuracy is not striking: Table 1 (Additional file 1). On the basis of these results TDI reduces significantly the diagnostic accuracy of dobutamine stress echocardiography whereas strain rate imaging equals the diagnostic accuracy but it does not improve it. Interestingly enough, the sensitivities and specificities of strain rate imaging are slightly different in the two papers even though the analysis was conducted in the very same set of patients. Moreover, since coronary angiography was performed in all patients, the diagnostic accuracy should have been calculated on this real gold standard instead of perfusion scintigraphy.

In a recent [33] article Marwick et al. questioned the hypothesis that false-negative results of dobutamine stress echocardiography reflect the underinterpretation of regional left ventricular function. On the opposite, the quantitative parameters such as strain rate and peak systolic strain rate were no different between false and true negative tests, suggesting that false-negative results are related to lack of ischemia in a functional sense. On the basis of this observation, quantitative markers are unlikely to increase the sensitivity of dobutamine stress echocardiography.

## Conclusions

The quantitative interpretation of stress echocardiography is not superior to expert wall motion assessment. Open issues in the quantitative analysis remain at stake: which technique to be employed among systolic velocities, strain and strain rate, the assessment of normality criteria of myocardial velocities and how to interpret their values, the management of patients with regional and global left ventricular dysfunction, the analysis of the apical segments, the complexity of the analysis in a real clinical environment, the applicability to unselected populations, its unsuitability to exercise, the most widely used stressor in the clinical practice [34]. What is presented as a breakthrough technology should have already answered to these issues and when exported into the clinical arena should have provided an incremental value to the established and more easily accessible methods. It is at this point that we are lost in clinical translation: authoritative journals provide data that cannot be transferred into the daily life of a busy stress echocardiographic laboratory, although the general message is optimistic and tend to ignore flaws and limitations of the technique [35,36]). The advantage/disadvantage balance of a new technology should clearly be stated. The potential advantages should always outweigh the disadvantages related to the higher costs and higher complexity of analysis. Perhaps, the shape of the quantitative technology to come has not been designed yet [37,38]. TDI is one of the tools in our hands but apparently this is not its time. At least not on the basis of this evidence.

## Supplementary Material

Additional File 1 Table 1.TDI vs visual assessment of myocardial ischemia during dobutamine stress echocardiography. Click here for file
